# Changes in the diversity and functionality of viruses that can bleach healthy coral

**DOI:** 10.1128/msphere.00816-24

**Published:** 2024-11-26

**Authors:** Zhengyi Zhang, Mengmeng Tong, Wei Ding, Shuikai Liu, Mui-Choo Jong, Ahmed A. Radwan, Zhonghua Cai, Jin Zhou

**Affiliations:** 1 Shenzhen Public Platform for Screening and Application of Marine Microbial Resources, Institute for Ocean Engineering, Shenzhen International Graduate School, Tsinghua University, Shenzhen, Guangdong Province, China; 2 Marine Ecology and Human Factors Assessment Technical Innovation Center of Natural Resources Ministry, Tsinghua Shenzhen International Graduate School, Shenzhen, Guangdong Province, China; 3 Shenzhen Key Laboratory of Advanced Technology for Marine Ecology, Institute for Ocean Engineering, Shenzhen International Graduate School, Tsinghua University, Shenzhen, Guangdong Province, China; 4 Ocean College, Zhejiang University, Zhoushan, Zhejiang, China; 5 Shenzhen Zhihai Ocean Biotechnology Co., Ltd., Shenzhen, Guangdong Province, China; 6 Institute of Environment and Ecology, Shenzhen International Graduate School, Tsinghua University, Shenzhen, Guangdong, China; 7 Genetics and Cytology Department, National Research Centre (NRC), Cairo, Egypt; University of Michigan, Ann Arbor, Michigan, USA

**Keywords:** coral microbiomes, coral bleaching, viruses, *Vibrio *phages

## Abstract

Coral microbiomes play a crucial role in maintaining the health and functionality of holobionts. Disruption in the equilibrium of holobionts, including bacteria, fungi, and archaea, can result in the bleaching of coral. However, little is known about the viruses that can infect holobionts in coral, especially bacteriophages. Here, we employed a combination of amplicon and metagenomic analyses on *Acropora muricata* and *Galaxea astreata* to investigate the diversity and functionality of viruses in healthy and bleached corals. Analysis showed that the alpha diversity of holobionts (bacteria, eukaryotes, zooxanthellae, and lysogenic and lytic viruses) was higher in bleached corals than that in healthy corals. Meanwhile, bleached corals exhibited a relatively higher abundance of specific viral classes, including Revtraviricetes, Arfiviricetes, Faserviricetes, Caudoviricetes, Herviviricetes, and Tectiliviricetes; moreover, we found that the expression levels of functional genes involved in carbon and sulfur metabolism were enriched. An increase in *Vibrio* abundance has been reported as a notable factor in coral bleaching; our analysis also revealed an increased abundance of *Vibrio* in bleached coral. Finally, bleached corals contained a higher abundance of *Vibrio* phages and encoded more virulence factor genes to increase the competitiveness of *Vibrio* after coral bleaching. In conclusion, we attempted to understand the causes of coral bleaching from the perspective of phage–bacteria–coral tripartite interaction.

**IMPORTANCE:**

Viruses, especially bacteriophages, outnumber other microorganisms by approximately 10-fold and represent the most abundant members of coral holobionts. Corals represent a model system for the study of symbiosis, the influence of viruses on organisms inhabiting healthy coral reef, the role of rapid horizontal gene transfer, and the expression of auxiliary metabolic genes. However, the least studied component of coral holobiont are viruses. Therefore, there is a critical need to investigate the viral community of viruses, and their functionality, in healthy and bleached coral. Here, we compared the composition and functionality of viruses in healthy and bleached corals and found that viruses may participate in the induction of coral bleaching by enhancing the expression of virulence genes and other auxiliary metabolic functions.

## INTRODUCTION

Corals, known as the “rainforests” of the ocean, boast high levels of biodiversity despite occupying less than 0.5% of the ocean floor but harboring approximately 25% of all marine species (1). Corals serve as crucial fishing grounds and tourist attractions for coastal nations, benefiting over 500 million people annually (2). However, due to marine pollution, poor land management, overfishing, and global climate change, the biodiversity and coverage of coral reefs have declined worldwide at unprecedented rates over the past few decades, including coral bleaching events (3, 4). The in-depth investigation of coral health has revealed the vital role played by members of the coral holobionts, including endosymbiotic zooxanthellae, bacteria, archaea, and fungi, and their importance in understanding the environmental adaptability, regulatory mechanisms, and nutritional needs of coral (5). The bacteria, archaea, and fungi living within the tissues, skeleton, and surface mucus layer of coral serve many functions associated with biochemical cycles, including nitrogen fixation, digestion of organic matter, and photosynthesis (6, 7). Previous studies confirmed that partnerships between microbial symbionts can provide a wide range of beneficial functions within coral holobionts and that the functional activity of microbes may mitigate or even prevent disease occurrence (such as bleaching) by supporting the internal equilibrium between the host, Symbiodiniaceae, and bacteria (8, 9). Conversely, microbial dysbiosis is known to disrupt photosynthetic stability and finely tuned nutrient exchange between corals and their dinoflagellate partners, thus leading to the breakdown of photosymbiosis and subsequent bleaching (10, 11).

Compared to bacteria associated with coral holobionts, very little is known about the viruses that inhabit coral. Marine viruses are morphologically and genetically diverse. In their simplest form, viruses consist of a protein capsid that encapsulates their genetic material which can be either DNA or RNA (12). Corals harbor a diverse and abundant virome, and over 60 viral families have been identified in corals worldwide (13). A small number of viral families that belong to three major viral lineages are ubiquitous to corals and are predicted to infect coral cells as well as Symbiodiniaceae, bacteria, and archaea (12, 14). Symbiodiniaceae-infecting viruses could conceivably contribute to coral bleaching and mortality; however, in this study, we predominantly focus on viruses that could infect bacteria. Previous research has reported the increased abundance of viral particles during stress events or bleaching episodes (15–17). In essence, symbiosis thrives within a homeostatic environment in healthy coral and relies on intricate symbiotic interactions to sustain coral health. The interplay between bacteria and their viruses assumes a pivotal role in maintaining microbial diversity, as well as regulating chemical composition *via* virus-encoded auxiliary metabolic genes (vAMGs) within natural ecosystems (18, 19).

Viruses rely on host cells for their replication (20), and their replication strategies typically manifest in two different lifestyles: lysis and lysogeny, each exerting distinct effects on the composition and functional characteristics of the microbial community (21). Lytic viruses can influence the composition of microbial populations, facilitating the transfer of organic matter from cells to the dissolved organic matter pool (known as a viral shunt) and the classical food web (known as a viral sweep) (22). Moreover, lytic viruses tend to thrive in populations with a higher host density, such as organisms experiencing algal blooms (Kill-the-Winner) (12). Conversely, lysogenic viruses tend to encode vAMGs to bolster host defense systems, enhancing host survival advantages and establishing long-term symbiosis with their hosts (Piggyback-the-Winner) (23). Nevertheless, our understanding of the biodiversity and potential functionality of viruses within coral holobionts remains in its infancy. Consequently, a comprehensive investigation into how viruses replicate in response to the connection between coral health and bleaching is imperative. Furthermore, it is important to ascertain whether these viruses can infect bacteria to mediate coral bleaching.

Based on the specific characteristics of viruses and coral symbiotes, we hypothesized that viruses would exhibit wide diversity, composition, and replication strategies when compared between healthy and bleached corals and that viruses may induce bleaching in coral by altering the functionality of symbiotic microbes *via* vAMGs. To test this hypothesis, we conducted field sampling and indoor analysis using corals acquired from the South China Sea (SCS). We specifically chose the SCS because it has an area of coral cover comparable to the Great Barrier Reef (24). Moreover, the abundance of bleached coral is gradually increasing, thus representing a suitable resource from which to acquire natural samples for ecological research. The specific role of virus-dominated holobionts in the bleaching of coral remains poorly understood although changes in the composition of bacterial communities have been frequently associated with coral bleaching in the SCS (25). Therefore, we collected specimens of healthy and bleached coral from two different species (*Acropora muricata* and *Galaxea astreata*) and performed amplicon and metagenomic analyses. We aimed to identify differences between healthy and bleached corals in terms of viral diversity, replication strategy, and functional potential. Our overall aim was to verify our hypothesis and enrich our understanding of coral virus ecology.

## MATERIALS AND METHODS

### Sample collection and DNA extraction

During the sampling process, the physical and chemical environment of the seawater were analyzed by YSI (Hydrolab DS5, HACH, USA) and Flow Injection Analysis (IFCB 140, McLANE, USA). To identify the coral morphologically, we referred to skeletal and structure information provided by the Corals of the World website (http://www.coralsoftheworld.org). The mean salinity of the sampling sites was 26.3‰ ± 0.3‰, the mean temperature was 26.9 ± 0.2℃, the transparency was 3.84 ± 0.12 m, and the pH was 8.11 ± 0.10. The collected coral colonies were identified as *Acropora muricata* and *Galaxea astreata*.

Specifically, we collected 40 coral fragments (2 species × 2 state ×10 samples) from a coral reef in Sanya, Hainan Island, China (109.292°E, 18.123°N) in July 2023. The field samples originated from four groups (HA: healthy tissues from *Acropora muricata*; BA: bleached tissues from *Acropora muricata*; HG: healthy tissues from *Galaxea astreata*; and BG: bleached tissues from *Galaxea astreata*), and each group contained 10 fragments of biological replicates. Coral fragments were obtained by scuba divers with hammers and chisels. Samples of coral tissue (approximately 2–4 cm in diameter) were collected from the outer edge of each coral (26).

All samples were immediately stored in liquid nitrogen, and DNA was extracted using the DNeasy PowerSoil kit (Qiagen, Hilden, Germany) according to the manufacturer’s instructions. The quality and concentration of DNA were determined by 1.0% agarose gel electrophoresis and a NanoDrop ND-2000 spectrophotometer (Thermo Scientific Inc., Waltham, USA); then, the DNA was stored at −80 ℃ to await analysis. Each sample was divided into three aliquots for sequencing of the bacterial 16S rRNA gene, eukaryotic 18S rRNA gene, and Symbiodinium ITS2 rRNA gene. In addition, two samples were randomly selected from each group to form a pool; four pools were generated from each group for metagenomic sequencing.

### High-throughput sequencing

In order to identify the diverse range of bacteria, eukaryotes, and Symbiodinium in corals, the bacterial we first amplified 16S rRNA genes by using two primers: 338F (5′-ACTCCTACGGGAGGCAGCAG-3′) and 806R (5′-GGACTACHVGGGTWTCTAAT-3′). Then, the eukaryotic 18S rRNA genes were amplified by 3NDF (5′-GGCAAGTCTGGTGCCAG-3′) and V4-euk-R2R (5′-ACGGTATCTRATCRTCTTCG-3′). Finally, the ribosomal Internal Transcribed Spacer 2 (ITS2) RNA gene of Symbiodinium was amplified by ITSintfor2 (5′-GAATTGCAGAACTCCGTG-3′) and ITS2-reverse (5′-GGGATCCATATGCTTAAGTTCAGCGGGT-3′). These three PCR reactions have been described in a previous pipeline (26–28). All amplicons were sequenced on the illumina HiSeq 2500 platform. For high-throughput sequencing of the metagenome, we performed shotgun-metagenomic sequencing for the four samples of each group with the Illumina NovaSeq 6000 PE150 platform (MAGIGENE, Guangdong, China).

### Amplicon sequencing analysis

Sequence analysis of the analysis of the 16S rRNA gene, 18S rRNA gene, and ITS2 rRNA gene was completed with QIIME 2v 2023.7 and its plug-in DADA2, including quality control, along with feature table and representative sequence generation. Microbial diversity was estimated by calculating alpha diversity (Chao1 index), and community composition was estimated by beta diversity (Bray–Curtis distance) based on the q2-diversity pipeline within QIIME2 (29). The taxonomic assignment of bacteria and eukaryotes was performed by comparison with the SILVA SSURef NR99 version 138.1 database (30), and the taxonomic assignment of the Symbiodinium clade was performed by comparison against the symClade database (https://github.com/reefgenomics/SymPortal_framework/tree/master/symbiodiniaceaeDB) with blastn version 2.13.0 (ftp://ftp.ncbi.nlm.nih.gov/blast/executables/blast+/LATEST) using an *e*-value of 1*e*^−5^. All information recovered for the 16S rRNA gene, 18S rRNA gene, and ITS2 rRNA in this study is given in Tables S1 to S3, including sample name, sample grouping, amplicon sequence variants (ASVs), absolute abundance of the feature-table, and species classification.

### Recovery and taxonomic assignment of metagenome-assembled genomes

Raw reads were first trimmed by Trimmomatic version 0.39 with default parameters (31), and then clean reads from each group were co-assembled by MEGAHIT version 1.2.9 with preset meta-large parameters (32). Contigs ≥ 1 kb were imported into metaWRAP version 1.3.2 using a binning module with metabat2, maxbin2, concoct, and Bin_refinement module parameters (-c 50 x 10) (33). The bin sets generated contained 5257 MAGs and were further de-replicated with dRep version 3.2.2 with strict parameters (-comp 50 -con 10 -sa 0.95 -nc 0.3) (34). The final data set included 127 microbial operational taxonomic units (mOTUs). The taxonomy of representative genomes from the mOTUs was assigned using GTDB-tk version 2.1.1 based on the R214 version of the Genome Taxonomy Database (GTDB) release data, and the classification results were further refined by comparison with NCBI taxonomy with the metawrap classify_bins module (35). All mOTU information recovered in this study is provided in Table S4.

### Identification, clustering, and taxonomic classification of viral genomes

Metagenomic viral contigs (mVCs) were identified for all contigs ≥ 5 kb by utilizing (i) DeepVirFinder version 2020.11.21 with a score ≥0.9 and *P* ≤ 0.05 (36); (ii) VirSorter2 version 2.2.3 with a score ≥0.5 (37); (iii) VIBRANT version 1.2.1 with default parameters (38); and (iv) geNomad version 1.7.4 with default parameters (39). By combining these software packages, we identified mVCs that were further filtered for proviral regions by CheckV version 0.8.1 (40). All mVCs were then dereplicated and clustered at 95% average nucleotide identity and with an 85% alignment fraction of shorter sequences (https://github.com/snayfach/MGV), generating 2021 viral operational taxonomic units (vOTUs). The taxonomy of vOTUs was then annotated by geNomad version 1.7.4 at the family level. All vOTU information recovered by this analysis is given in Table S5.

### Clustering and annotation of the virulence factor gene and vAMGs

Prodigal v2.6.3 (-p meta) was used to predict open reading frames (ORFs) viral genomes (41), and all genes encoded by viruses were clustered at the nucleotide level using cd-hit version 4.8.1 with the strict parameters (-aS 0.9 c 0.95) to generate 27901 vPCs to represent functional viral structures (42). The virulence factor genes of *Vibrio* phages were identified by BLASTp searches against a specific database (http://www.mgc.ac.cn/VFs/) set to an -*e* value of 1*e*^−5^. vAMGs were identified by eggNOG-mapper version 2.1.12 set to an -*e* value 1*e*^−5^ (43), including the annotations of Kyoto Encyclopedia of Genes and Genomes (KEGG) orthology, KEGG pathway, and carbohydrate-active enzymes. All clustering and annotation of viral proteins included in this are given in Table S6.

### Lifestyle prediction and hosts for vOTUs

The life strategies of vOTUs were predicted by applying two pipelines: (i) VIBRANT version l.2.0 was used to infer temperate life strategies by identifying vOTUs that contained integrase genes (38, 40) and (ii) ORFs from all vOTUs were functionally annotated using eggNOG-mapper version 2.1.12 and sequences containing lysogeny-specific genes (i.e., integrases, recombinases.transposases, excisionases, CI/Cro repressor, and parAB) were selected and manually inspected (43). The vOTUs identified by the two pipelines were considered to be temperate, while others were considered to be lytic. Lifestyle predictions and hosts for vOTUs are given in Table S5.

### Calculating the normalized abundance of vOTUs, mOTUs, and vPCs

Clean reads from each sample were first mapped to representative genomes in the Coverm version 0.6.1 pipeline (https:// github. com/wwood/CoverM) pipeline with the following parameters: (i) mOTUs, using the Coverm genome module (parameters: -p bwa-mem --trim-min 0.10 --trim-max 0.90 --min-read-percent-identity 0.95 --min-read-aligned-percent 0.75 m rpkm) and (ii) vOTUs and vPCs, using the Coverm contig module (parameters: -p bwa-mem --trim-min 0.10 --trim-max 0.90 --min-read-percent-identity 0.95 --min-read-aligned-percent 0.75 m rpkm). RPKM (reads per kilobase per million mapped reads) values were used to represent the normalized abundances of mOTUs, vOTUs, and vPCs. The normalized abundance of vOTUs, mOTUs, and vPCs is given in supplementary material (Tables S4 to S7).

### Statistical analyses

All statistical analyses were performed using the R platform v 4.3.1 (https://www.rproject.org/). Alpha and beta diversity analyses (including Chao1 index and PCoA based on the Bray–Curtis dissimilarity matrix) of coral holobionts from the amplicons and microorganisms from the metagenome were conducted using vegan and ggplot2 packages in R. Alpha and beta diversity weer compared with Wilcox.test (stat_compare_means function) and Permutational analysis of variance (PERMANOVA) (Adonis function). Differences in viral classification and functionality between health and bleached coral were determined by the Wilcox test.

## RESULTS

### Overview of the diversity holobionts other than viruses

**Fig 1:**
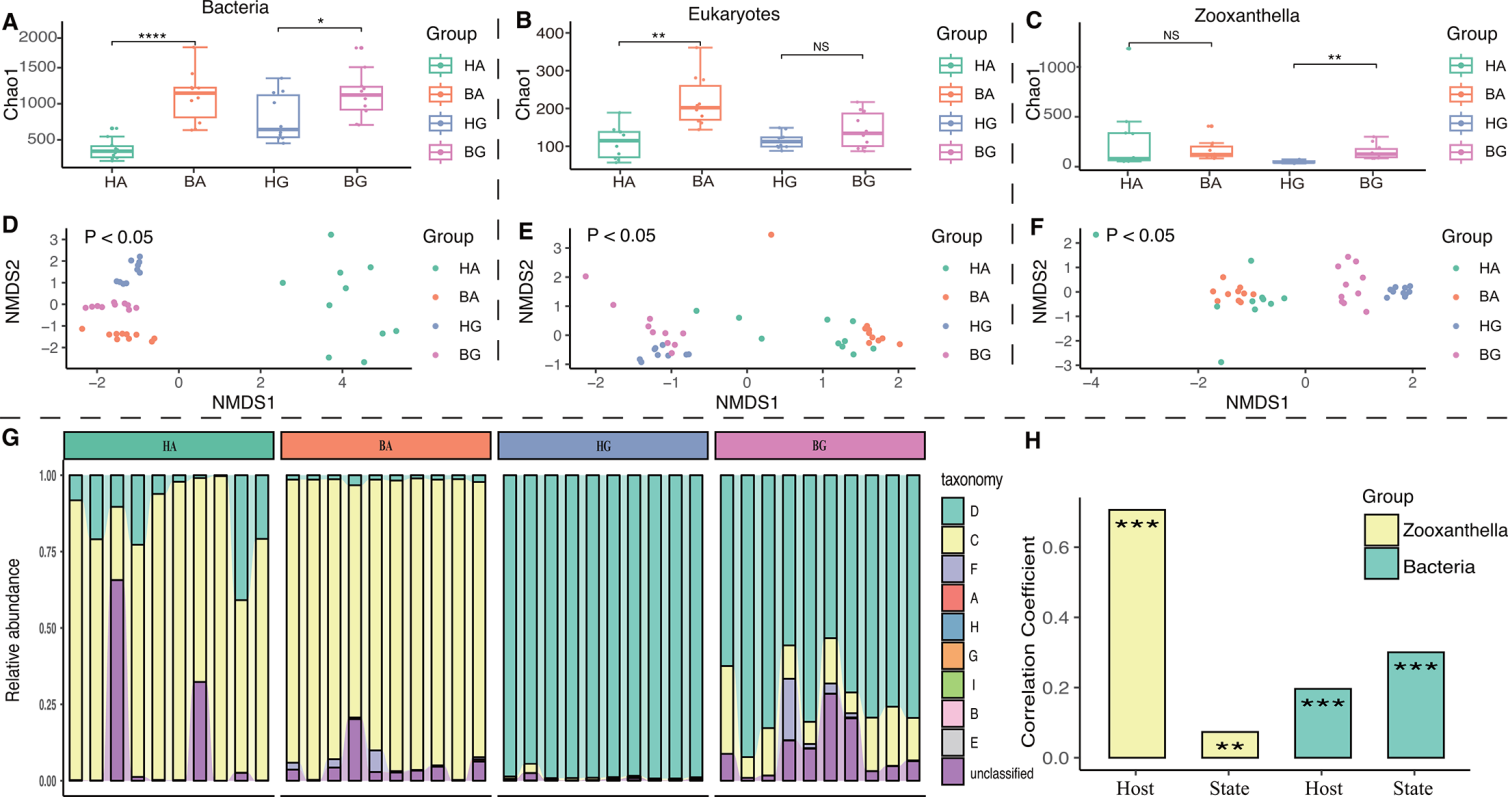
Overview of bacteria, eukaryotes, and zooxanthella in coral holobionts by amplicon sequencing. (**A**) The Chao1 index of bacterial diversity based on sequencing of the 16S rRNA gene, (**B**) the 18S rRNA gene, and (**C**) the ITS rRNA gene across different groups (HA, BA, HG, and BG). NS indicates not significant, * indicates *P* < 0.05, ** indicates *P* < 0.01, and **** indicates *P* < 0.0001. (**D–F**) NMDS based on Bray–Curtis dissimilarity revealed the community structure of coral holobionts and identified differences based on PERMANOVA. (**G**) The relative abundance of zooxanthellae at the clade-level. (**H**) Effects of host and state on the community diversity of zooxanthellae and bacteria. ** indicates *P* < 0.01, *** indicates *P* < 0.001.

In addition to viruses, we detected a wide diversity of bacteria, eukaryotes, and zooxanthella when comparing healthy and bleached corals (*Acropora muricata* and *Galaxea astreata*) by sequencing amplicons generated from three target genes. Rarefaction curves reached saturation with increasing numbers of sequences, indicating that the alpha diversity of bacteria, eukaryotes, and zooxanthella had been captured (Fig. S1). When considering the alpha diversity of holobionts based on Chao1, it was evident that bleached corals had a higher alpha diversity than that in healthy corals (HA vs BA and HG vs BG for bacteria, HA vs BA for eukaryotes, HG vs BG for zooxanthella) (*P* < 0.05) (Fig. 1A through C). However, there was no significant difference between two groups (HG vs BG for eukaryotes, HA vs BA for zooxanthella) (Fig. 1B and C). With regard to beta diversity of the holobionts, Bray–Curtis analysis revealed significant differences between the four coral groups (*P* < 0.05) (Fig. 1D through F). In addition, the contribution made by zooxanthella to diversity was predominantly driven by the host (*R*^2^ = 0.7, *P* < 0.05) rather than a healthy state (*R*^2^ = 0.07, *P* < 0.05) (Fig. 1H). For example, *Acropora muricata* was dominated by clade C, while *Galaxea astreata* was dominated by clade D (Fig. 1G). However, the contribution to bacterial diversity was predominantly driven by a healthy state (*R*^2^ = 0.3, *P* < 0.05) rather than the host species (*R*^2^ = 0.19, *P* < 0.05) (Fig. 1H) (25, 44); our present analysis confirmed this previous finding. On this basis, our next step was to investigate the diversity of viruses in coral after bleaching.

### Viral community profiles in coral holobionts as determined by metagenomic sequencing

**Fig 2:**
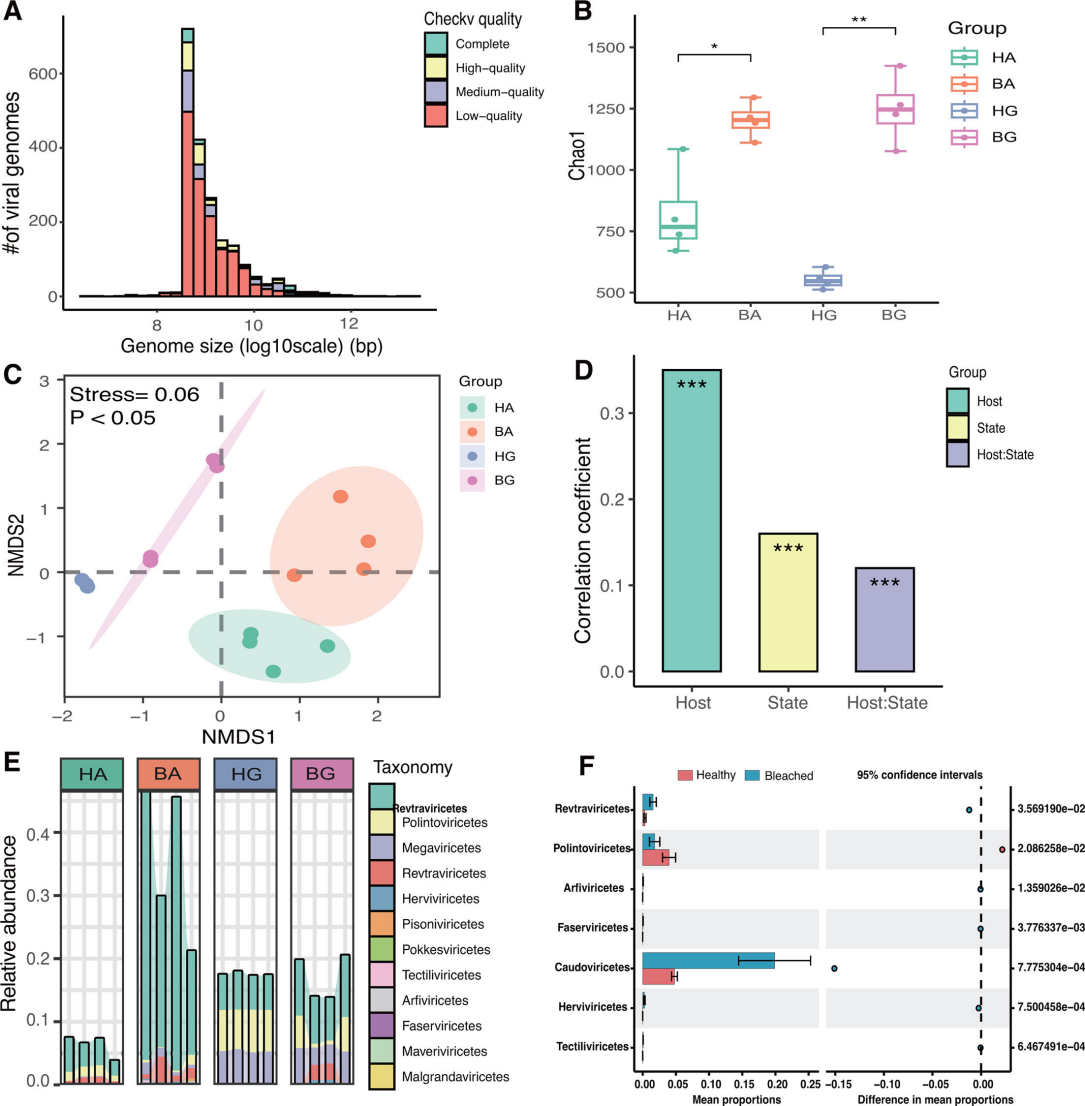
Overview of the viral genome in coral recovered by metagenomics. (**A**) Histogram showing the distribution of viral genome size (log10 scale) and quality. (**B**) Chao1 index of viral alpha diversity across different groups, * indicates *P* < 0.05, and ** indicates *P* < 0.01. (**C**) NMDS analysis of viral communities based on Bray–Curtis dissimilarity; PERMANOVA was applied to detect differences between groups. (**D**) Bar plot showing the contribution of host, state, and host-state interactions to virus diversity. (**E**) The relative abundance of viral taxa across four coral groups based on RPKM values. (**F**) Differences in the relative abundance of viruses at the family level between healthy and bleached corals.

To further investigate the changes in the viral diversity of coral after bleaching, we next conducted in-depth metagenomic sequencing. The study data set featured 16 metagenome pools from four groups of coral (HA, BA, HG, and BG), amounting to approximately 320 GB of sequencing data. With regard to the viral genomes retrieved by metagenomic sequencing, 2,082 metagenomic viral contigs (mVCs) were identified by various viral identification methods. These contigs were further clustered at the species level with a 95% mean nucleotide identity threshold, resulting in 2021 vOTUs for subsequent analysis. Of the vOTUs assembled by analysis of the metagenomic library, 88 were complete, 215 were of high quality, 260 were medium quality, and 1,458 were low quality. The lengths of the viral contigs ranged from 0.658 to 570 kb (Fig. 2A; Table S5).

Next, we considered the alpha diversity of viruses by calculating the Chao1 index; analysis revealed that the alpha diversity of bleached *Acropora muricata* and *Galaxea astreata* was significantly higher than that of healthy corals (*P* < 0.05) (Fig. 2B), consistent with the pattern observed for bacterial diversity. Next, the patterns of beta diversity between viral contigs were assessed using Bray–Curtis distances. PERMANOVA revealed significant partitioning of distance variance across the four coral groups (*P* < 0.05) (Fig. 2C). The pattern of viral community structure, based on Bray–Curtis distances, was consistent with the pattern of bacterial beta diversity. The contribution to viral diversity was predominantly driven by the host (*R*^2^ = 0.35, *P* < 0.05) rather than a healthy state (*R*^2^ = 0.16, *P* < 0.05); the lowest contribution was identified as host and state interaction (*R*^2^ = 0.12, *P* < 0.05) (Fig. 2D).

With regard to the taxonomic assignment of viruses, only 893 of the 2,021 vOTUs were annotated as viral taxa (44.19%), representing 4 phyla, 7 classes, 9 orders, and 12 families (Table S5). Overall, the top four most abundant viral families, in descending order, were Caudoviricetes (12.33%), Polintoviricetes (2.89%), Megaviricetes (2.62%), and Revtraviricetes (0.95%) (Fig. 2E). Additional analysis revealed that bleached coral exhibited significantly higher proportions of Revtraviricetes, Arfiviricetes, Faserviricetes, Caudoviricetes, Herviviricetes, and Tectiliviricetes, but lower levels of Polintoviricetes, when compared to healthy coral at the family level (*P* < 0.05) (Fig. 2F). In summary, our analyses revealed that viral diversity increases after coral bleaching and is mainly enriched by Revtraviricetes and Arfiviricetes.

### Viral lifestyle

**Fig 3:**
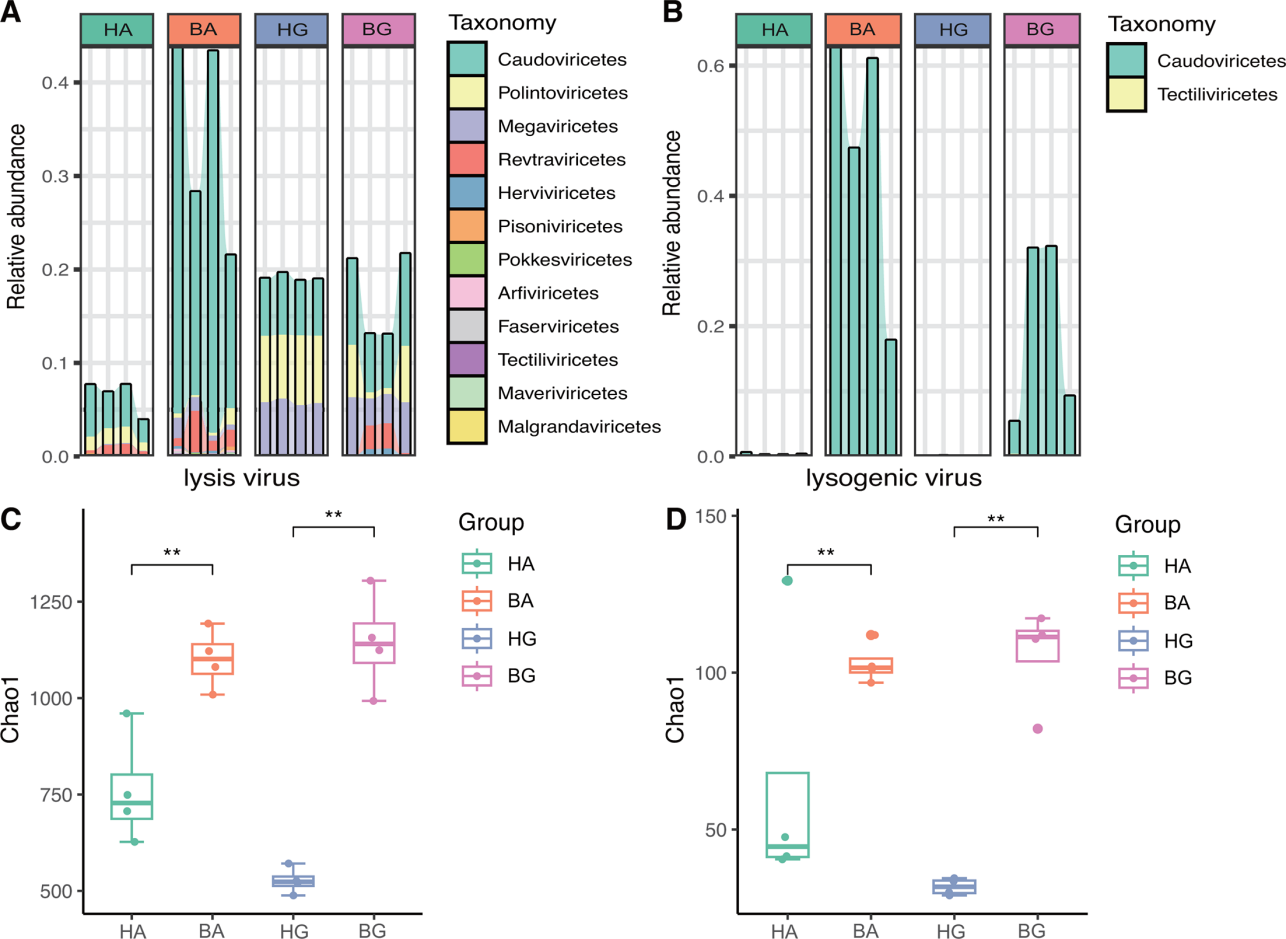
Changes in the lifestyles of viruses among four coral groups. The relative abundances of lytic (**A**) and lysogenic (**B**) viruses in terms of taxonomy. The alpha diversity based on Chao1 of lytic (**C**) and lysogenic (**D**) viruses, ** indicates *P* < 0.01.

To investigate the effects of viral replication strategies on coral bleaching, we next predicted their viral lifestyle. Out of the 2021 vOTUs identified, 161 were determined to be lysogenic (7.97%), while the remaining putative viruses were categorized as lytic (92.03%). Of the lytic viruses, the top four relative abundances at the family level, in descending order, were Caudoviricetes (11.87%), Polintoviricetes (3.13%), Megaviricetes (2.85%), and Revtraviricetes (1.02%) (Fig. 3A). Furthermore, alpha diversity, as indicated by the Chao1 index, was significantly higher in bleached coral when compared to healthy coral (*P* < 0.05) (Fig. 3C). With regard to lysogenic viruses, the relative abundance at the family level, in descending order, was dominated by Caudoviricetes (16.74%) followed by Tectiliviricetes (0.05%) (Fig. 3B). Similarly, alpha diversity, as indicated by the Chao1 index, was significantly higher in bleached coral than in healthy coral (*P* < 0.05) (Fig. 3D). In summary, lysogenic and lytic viruses in bleached coral exhibited higher abundances of Caudoviricetes and greater alpha diversity, based on the Chao1 index, when compared to healthy coral.

### Virus-host linkage prediction

**Fig 4:**
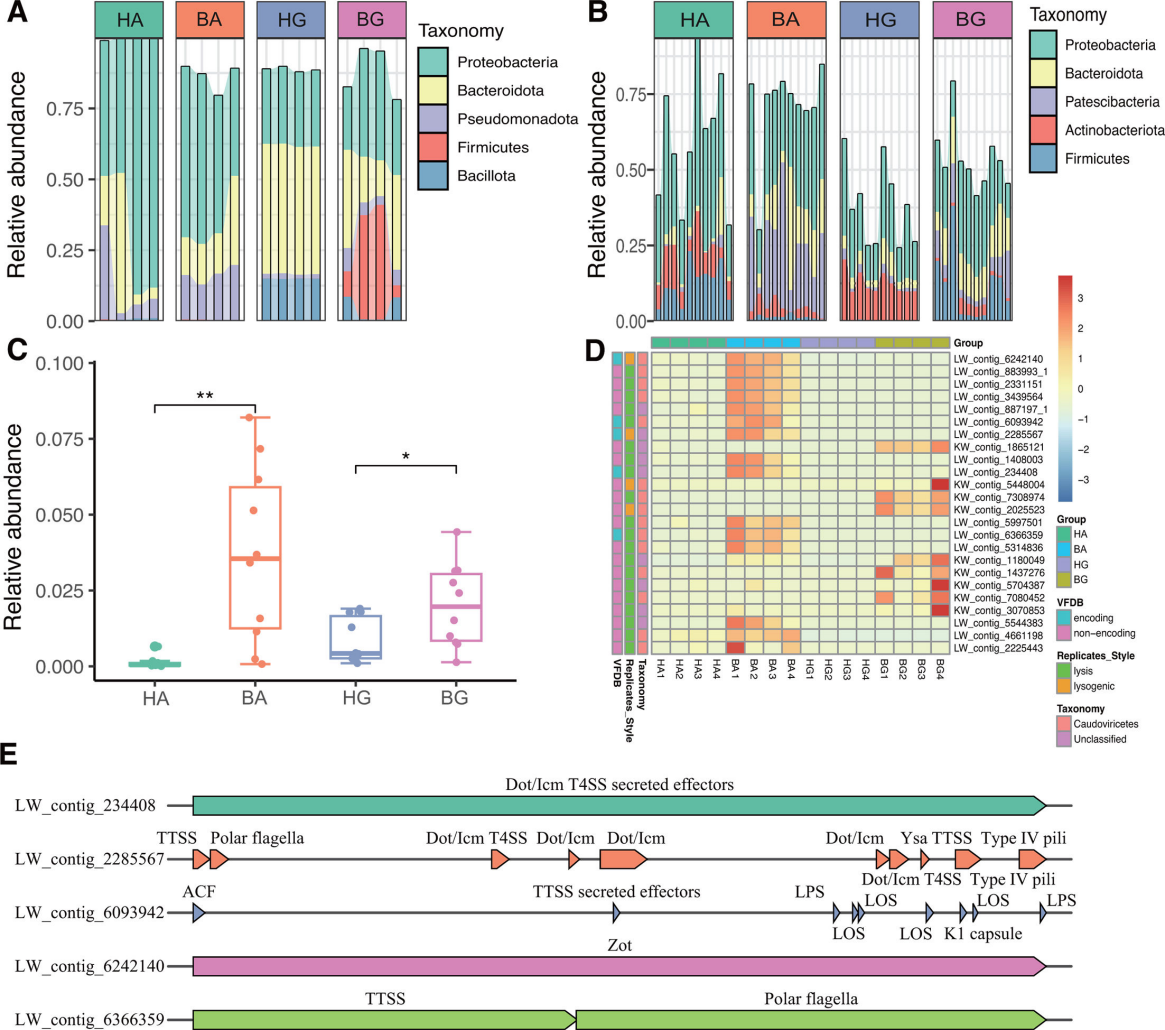
Virus-host linkage prediction among four coral groups. (**A**) The relative abundance of viruses as predicted by metagenomic sequencing. (**B**) Relative abundance based on 16S rRNA gene sequencing. (**C**) Differences in the relative abundance based on 16S rRNA gene sequencing of *Vibrio*, * indicates *P* < 0.05, and ** indicates *P* < 0.01. (**D**) Relative abundance, replication strategies, and virulence genes of *Vibrio* phages. (**E**) Representative arrow maps of the detected vAMGs associated with virulence factors.

To investigate how viruses bleach coral by infecting bacteria, we performed virus–bacterial linkage prediction. Putative viral hosts were successfully assigned to 284 viral genomes. The most frequently predicted host for the viral genomes from four coral groups were Proteobacteria (44.41%) and Bacteroidota (26.62%), followed by Pseudomonadota (8.69%), Firmicutes (5.72%), and Bacillota (5.06%) (Fig. 4A). In addition, the most frequent host, based on 16S rRNA gene sequencing, were Proteobacteria (26.36%) and Bacteroidota (8.22%), followed by Patescibacteria (7.77%), Actinobacteriota (6.77%), and Firmicutes (6.39%) (Fig. 4B). Our analyses revealed an abundance of viral taxa followed by bacterial taxa, including Pseudomonadota and Firmicutes (Fig. 4A and B).

It has been extensively reported that coral bleaching may be related to an increased abundance of *Vibrio* (25). Our analysis revealed that the relative abundance of *Vibrio* in the two different hosts was significantly higher in bleached corals than in healthy corals, as determined by 16S rRNA sequencing (*P* < 0.05) (Fig. 4C). Then, we predicted 24 phages related to *Vibrio* by virus-host linkage; of these phages, 15 putative viruses were known viral classifications (Duplodnaviria, Heunggongvirae, Uroviricota, and Caudoviricetes). The other nine putative viruses were unclassified and may represent new candidate *Vibrio* phages. Only four of the 24 *Vibrio* vOTUs (16.67%) were identified as lysogenic; the other 20 viruses were identified as lytic (83.33%) (Fig. 4D). In addition, analysis showed that the relative abundance of *Vibrio* phage in the two different hosts was significantly higher in bleached corals than in healthy corals (*P* < 0.05) (Fig. 4D). Next, we performed functional annotation on the virulence factor genes carried by *Vibrio* phages. We identified five *Vibrio* viruses that encoded virulence factors, including LW_contig_234408 (Dot/Icm T4SS secreted effectors), LW_contig_2285567 (TTSS, Polar flagella, Dot/Icm T4SS, Dot/Icm, Ysa TTSS, Type IV pili), LW_contig_6242140 (Zot), LW_contig_6093942 (ACF, TTSS secreted effectors, LPS, LOS, LOS, Capsule, K1 capsule, LOS, LPS), and LW_contig_6366359 (TTSS, Polar flagella) (Fig. 4E). The virulence factors encoded by these viruses are likely to improve the survival competitiveness of *Vibrio* and, therefore, influence the bleaching process in coral.

### Metabolic profiles of vAMGs

Next, we conducted a comprehensive investigation of vAMGs, especially those related to the cycling of elements. Overall, 1,311 of 27,901 vPCs (~ 4.69%) were identified as vAMGs, covering 11 metabolic classes. Of these, coral viruses tended to encode AMGs for “metabolism of cofactors and vitamins” (149 out of 1,311), “amino acid metabolism” (106 out of 1,311), “carbohydrate metabolism” (85 out of 1,311), “energy metabolism” (62 out of 1,311), “nucleotide metabolism” (57 out of 1,311), “glycan biosynthesis and metabolism” (40 out of 1,311), “metabolism of other amino acids” (31 out of 1,311), “lipid metabolism” (29 out of 1,311), “biosynthesis of other secondary metabolites” (24 out of 1,311), “metabolism of terpenoids and polyketides” (23 out of 1,311), and “cenobiotics biodegradation and metabolism” (18 out of 1,311) (Table S6).

**Fig 5:**
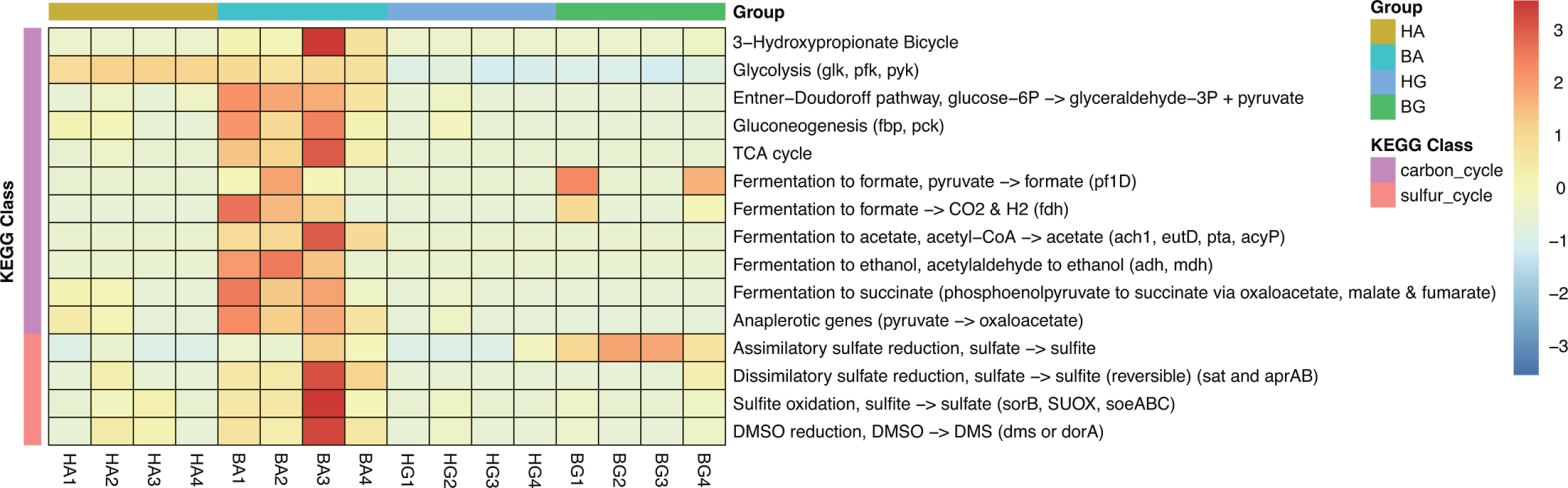
Heatmap representing the abundance of virus-encoded genes enriched in KEGG pathways associated with element cycling, including carbon and sulfur metabolism.

To investigate the potential functional role of viruses on symbionts on coral, we selected carbon (C), nitrogen (N), phosphorus (P), and sulfur (S) as examples and investigated all vAMGs related to their metabolism; 21 vAMGs were classified as C metabolism genes, featuring 11 metabolism pathways, including 3-Hydroxypropionate Bicycle and Glycolysis (*glk*, *pfk*, *pyk*) (Fig. 5). In addition, we investigated and refined carbohydrate-active enzymes (CAZymes) in vAMGs, including auxiliary activities (AA) from AA10 (1), carbohydrate-binding modules (CBMs) from the CBM48 (4) and CBM73 (2) families, as well as glycosyl hydrolases (GHs) from the GT1 (1), GT4 (3), GT13 (4), GT14 (1), GH19 (1), GT20 (1), GH23 (4), GH26 (1), GH27 (1), GT29 (1), GT30 (1), GT51 (1), and GH77 (1) families (Table S6). With regard to S metabolism, only five vAMGs were annotated, including four important S metabolism pathways. Of these, vAMGs related to assimilatory sulfate reduction, dissimilatory sulfate reduction, sulfite oxidation, and DMSO reduction (Fig. 5). All of the virus-encoded genes shown to be enriched by KEGG pathway analysis and associated with element cycling were detected in genes encoding bacteria (Fig. S2). However, the vAMGs associated with N and P were not detect by KEGG orthology gene. Analysis showed that the viruses present in bleached coral exhibited a higher potential to harbor vAMGs related to C and S metabolism when compared to healthy corals (Fig. 5).

## DISCUSSION

Coral reefs thrive in nutrient-poor shallow waters, serving as biodiversity and productivity hotspots, yet facing threats from anthropogenic disturbances, including coral bleaching (12). Within corals, the microbiota comprises a diverse array of microbial organisms, including archaea, bacteria, eukaryotes, and viruses (45, 46). These components collectively play pivotal roles in regulating the physiology of holobionts. The microbiota fulfills a variety of functions within coral holobionts, including nutrient acquisition, protection against pathogens, conditioning of the immune system, and enhancement of stress tolerance (47, 48). Given the abundance of bacterial hosts within coral holobionts, bacteriophages (viruses that can infect bacteria) have emerged as a significant microbial component (49). However, the relationships between virus–bacteria interactions and their potential subsequent effects on coral bleaching remain unclear. In this context, we investigated the diversity and functional disparities of virus-dominated holobionts between two species of healthy and bleached corals. Our approach involved high-depth amplicon and metagenomic sequencing and aimed to elucidate the potential connection between coral bleaching and viral dynamics.

Both the host (*Acropora muricata* and *Galaxea astreata*) and state (the transition from eubiosis to dysbiosis) are known to reshape the microbial diversity in coral. Our analysis revealed that the alpha diversity, based on the Chao1 index, for coral-associated holobionts (bacteria, eukaryotes, endolithic algae, and viruses) was significantly higher in coral following bleaching (Fig. 1A through C and 2B). The high diversity of holobionts in bleached coral may be explained by the intermediate disturbance hypothesis of the external environment (50). Bleached corals are known to contain a high abundance of decomposed tissue and also release more nutrients; moreover, some pioneer species, such as *Vibrio*, employ R-selected life history strategies by competing with other bacteria. In our current research, we found a higher abundance of *Vibrio* in bleached coral (Fig. 4C). This observation was supported by the fact that bleached corals are known to exhibit an increased level of community diversity, along with a reduction in mutualistic and key bacterial symbionts, such as Endozoicomonas species (51). Conversely, in bleached coral, there is a shift toward an increase in opportunistic bacteria and potential pathogens, such as *Vibrio* species (10, 25). In addition, our research revealed that beta diversity differed significantly between the four groups of coral. This finding indicated that the microbial composition of coral symbiotes (including viruses) was co-influenced by host type and health state. However, the influence of host and health state on the diversity of different members of holobionts exhibited different patterns. For example, the contribution of bacterial diversity was predominantly driven by state rather than the host (Fig. 1H). However, for zooxanthellae and viruses, diversity was predominantly driven by the host rather than the state of health (Fig. 1H and 2D). It is possible that viruses and zooxanthellae exhibit a closer co-evolutionary relationship with their coral hosts when compared with bacteria.

Coral viruses appear to represent a “solution” to the paradox created by nutrient-poor environments and highly productive ecosystems by infecting and lysing bacteria (12). Our analysis revealed that most of the viruses found in corals are lytic (Fig. 3A and B) and that the alpha diversity of lytic and lysogenic viruses increased significantly in coral following bleaching (Fig. 3C and D). In general, viruses can induce the release of large amounts of dissolved organic carbon (DOC) by lysing bacteria; this organic carbon can then be taken up and used by symbiotic microbes within the coral (52). On the one hand, bleached coral exhibited a higher microbial alpha diversity. Similarly, the alpha diversity of lytic viruses also increased in bleached coral, maintaining the relative higher biodiversity of holobionts by releasing more DOC. On the other hand, coral “bleaching” specifically describes the gross loss of Symbiodinium algal cells and chlorophyll from a host (53). If a bleached host does not recover its symbiotic community within weeks to months after bleaching, then disease and partial or total host mortality can occur (54). During this period, all holobionts experience extreme external environmental pressure. We hypothesize that the abundance of lysogenic viruses in coral increases significantly following bleaching to help the host to survive, thus creating a response strategy to various environmental changes.

Shifts in the composition and relative abundance of a viral community are likely to correlate with differences in coral health. Our results show that bleached coral featured a greater abundance of Revtraviricetes, Arfiviricetes, Faserviricetes, Caudoviricetes, Herviviricetes, and Tectiliviricetes at the family level when compared with healthy coral (Fig. 2E). These viruses are often bacteriophages and act particularly on certain pathogenic or conditional pathogenic bacteria. For example, we detected a significant increase in the relative abundance of *Vibrio* and their phages in bleached coral (Fig. 4D). The abundance of *Vibrio* phages carrying vAMGs associated with virulence factors was also significantly increased in bleached coral (Fig. 4E). Species of *Vibrio*, such as *Vibrio Mediterraneae*, are well-known coral pathogens and can cause disease in coral and induce the death of scallops and giant scallops (55, 56). Several *Vibrio* species are known to increase their virulence potential to improve their competitiveness *via* viruses encoding more virulence factors (10). Thus, species of *Vibrio* can occupy a dominant ecological niche and that viruses can enhance the survival competitiveness of *Vibrio via* vAMGs. This may explain how coral bleaching is associated with the indirect pathogenic potential of viruses. Furthermore, most *Vibrio* phages are lytic and can kill more dominant species, such as *Vibrio.*

Viruses of holobionts can be indirectly involved in the metabolism of elements by coral. Previous research demonstrated that coral-associated bacteria play important roles in maintaining the dynamic homeostasis of holobionts, forming a network of connections that include carbon uptake, sulfur cycling, and production of antimicrobial agents, thereby facilitating the biological control of pathogens (57). Similarly, viruses related to bacteria also appear to help maintain the homeostatis of symbionts *via* vAMGs associated with carbohydrate and sulfur metabolism. In zooxanthellae, photosynthesis provides a major source of carbon for microbial development. When deprived of autotrophic-derived substrates during bleaching, the microbial community potentially shifts toward other forms of nutrient acquisition. Our analysis found that the activity of pathways involving vAMGs associated with carbon fixation (3-hydroxypropionate bicycle) increases during the bleaching of coral; this appears to be a manifestation of microbial adaptation. In addition, nutrient interactions within holobionts can change dramatically during periods of stress, with coral microbiomes in bleached coral exhibiting the increased expression of genes associated with the metabolism of carbohydrates and sulfur (17, 58). Our analysis showed that viral vAMGs are involved in carbohydrate and sulfur metabolism, especially in bleached corals (Fig. 5). This may be due to a stress-induced shift in microbiome composition (25) toward an opportunistic community that takes advantage of destabilization in the utilization pathways of carbon and sulfur.

While our research focused on investigating relative abundance to ascertain the heritability of the coral microbiome between healthy and bleaching states in two species of coral, it is imperative that we acknowledge certain limitations in our study. Typically, microbiome heritability is inferred from relative abundance data, for which taxon-specific abundances are expressed as proportions of the total microbial abundance in a given sample. However, research conducted by Marjolein et al. previously revealed that relying solely on relative abundance data may misrepresent microbiome heritability (59). Three key issues arise when using relative abundances to estimate microbiome heritability: (i) interdependency between taxa can lead to imprecise heritability estimates, (ii) large sample sizes lead to high false discovery rates, and (iii) microbial co-abundances result in biased heritability estimates (59). Hence, it is imperative that future investigations incorporate absolute abundance measurements to provide a more comprehensive understanding of heritability in the coral microbiome.

### Conclusion

Coral microbiomes play a pivotal role in sustaining the health and functionality of holobionts. Holobionts can transition from eubiosis, indicative of a healthy state, to dysbiosis, signaling an unhealthy and disrupted condition, particularly in the presence of deteriorating environmental circumstances. In this research, we considered viruses (the most prevalent biological entities within holobionts) as a specific model and detected a relatively higher abundance of Revtraviricetes, Arfiviricetes, Faserviricetes, Caudoviricetes, and Herviviricetes in bleached coral, indicating that these species may represent important indicators of coral bleaching. Furthermore, bleached corals featured an increased abundance of *Vibrio* phages and expressed a greater number of virulence factor genes, as well as AMG-induced C/S metabolism disruption, thus suggesting a potential correlation between viral activity and coral bleaching. Our findings confirmed our hypothesis that viruses participate in shaping coral symbiotes by altering their composition and life history strategies and indirectly affect host health *via* the AMG pathway. Our study provides scientific data that enhance our understanding of viral microecological roles in coral symbiotes.

## Data Availability

Raw sequencing reads have been deposited in the National Center for Biotechnology Information (NCBI) Sequence Read Archive (SRA) with the accession number PRJNA1090147.

## SUPPLEMENTAL MATERIAL

Supplemental figures msphere.00816-24-s0001.docx Fig. S1 and S2.

Supplemental tables msphere.00816-24-s0002.xlsx Tables S1 to S7.

ASM does not own the copyrights to Supplemental Material that may be linked to, or accessed through, an article. The authors have granted ASM a non-exclusive, world-wide license to publish the Supplemental Material files. Please contact the corresponding author directly for reuse.
