# Supplementary material for: Changes in the diversity and functionality of viruses that can bleach healthy coral
Source: mSphere. 2024 Nov 26;9(12):e00816-24. doi: 10.1128/msphere.00816-24 (PMC11656804; doi:10.1128/msphere.00816-24)
Supplement: Supplemental figures — Fig. S1 and S2. [file msphere.00816-24-s0001.docx]

Supplementary Material

**Changes in the diversity and functionality of viruses that can bleach healthy coral**

Zhengyi Zhang^1,2,3^, Mengmeng Tong^4^, Mui-Choo Jong^5^, Ahmed A. Radwan^6^, Zhonghua Cai^1,2,3^, Jin Zhou^1,2,3*^

^1^Shenzhen Public Platform for Screening and Application of Marine Microbial Resources, Institute for Ocean Engineering, Shenzhen International Graduate School, Tsinghua University, Shenzhen 518055, Guangdong Province, P. R. China

^2^Marine Ecology and Human Factors Assessment Technical Innovation Center of Natural Resources Ministry, Tsinghua Shenzhen International Graduate School, Shenzhen 518055, Guangdong Province, P. R. China

^3^Shenzhen Key Laboratory of Advanced Technology for Marine Ecology, Institute for Ocean Engineering, Shenzhen International Graduate School, Tsinghua University, Shenzhen 518055, Guangdong Province, P. R. China

^4^Ocean College, Zhejiang University, Zhoushan 316021, PR China

^5^Institute of Environment and Ecology, Shenzhen International Graduate School, Tsinghua University, Shenzhen, Guangdong, China.

^6^Genetics and Cytology Department, National Research Centre (NRC), Cairo, 12622, Egypt

* Corresponding author: Jin Zhou

Email address: [zhou.jin@sz.tsinghua.edu.cn](mailto:zhou.jin@sz.tsinghua.edu.cn)

**Figure S1.** The rarefaction curves of bacteria, eukaryotes and zooxanthella among four coral groups (HA, BA, HG, and BG).

**Figure S2.** Heatmap represents the abundance of bacteria-encoded genes enriched in KEGG pathway associated with element cycle such as C and S metabolism. Red represents KEGG pathways that are also present in virus-encoded genes.
